# Outcomes of adults with community-acquired bacterial meningitis in the Netherlands: a prospective nationwide cohort study

**DOI:** 10.1016/j.lanepe.2025.101529

**Published:** 2025-11-14

**Authors:** Evelien H.G.M. Drost, Eva N. Schepers, Nora Chekrouni, Thijs M. van Soest, Diederik L.H. Koelman, Merijn W. Bijlsma, Matthijs C. Brouwer, Diederik van de Beek

**Affiliations:** Department of Neurology, Amsterdam Neuroscience, Amsterdam UMC, University of Amsterdam, Meibergdreef 9, Amsterdam, the Netherlands

**Keywords:** Bacterial meningitis, Streptococcus pneumoniae, Listeria monocytogenes, Streptococcus pyogenes, Neisseria meningitidis, Haemophilus influenzae, Neurological sequelae, Dexamethasone

## Abstract

**Background:**

Bacterial meningitis is associated with high rates of unfavourable outcome. We conducted a nationwide prospective study in the Netherlands to evaluate outcome in adults with bacterial meningitis, focusing on pathogen-specific differences, risk factors for unfavourable outcomes, and neurological sequelae.

**Methods:**

Adult patients with community-acquired bacterial meningitis confirmed by lumbar puncture were identified through the database of the Netherlands Reference Laboratory for Bacterial Meningitis and prospectively included. Neurological examinations were performed on admission and discharge by the treating physician, and outcome was assessed using the Glasgow Outcome Scale, with scores of 1–4 classified as unfavourable. Logistic regression was used to evaluate the association between potential predictors and outcome.

**Findings:**

Between January 1, 2006 and January 1, 2024, 2974 patients were included. *Streptococcus pneumoniae* was the most common pathogen (2029/2974; 68%), followed by *Neisseria meningitidis* (329/2974; 11%), *Listeria monocytogenes* (182/2974; 6%), *Haemophilus influenzae* (119/2974; 4%) and *Streptococcus pyogenes* (83/2974; 3%). Overall mortality was 17% (516/2974) and remained stable over 18 years (odds ratio per admission year 0·99 [95% CI 0·97–1·01]). Mortality was highest for *L*. *monocytogenes* (58/182; 32%), *S*. *pyogenes* (16/83; 19%), and *S*. *pneumoniae* (365/2029; 18%). Unfavourable outcome occurred in 1161/2974 patients (39%; 95% CI 37–41), and key predictors included advanced age, prolonged symptom duration, systemic or cerebral compromise, low CSF white-cell counts, and absence of adjunctive dexamethasone. Among survivors, neurological sequelae occurred in 1146/2088 patients (55%), including hearing impairment (692/2197; 31%) and cognitive impairment (481/2108; 23%), with highest rates following *S*. *pneumoniae* (881/1419; 62%), and *S*. *pyogenes* (44/59; 75%).

**Interpretation:**

Mortality and morbidity from bacterial meningitis remain high, especially in *L*. *monocytogenes*, *S*. *pyogenes*, and *S*. *pneumoniae* infections. These findings highlight an urgent need for enhanced vaccination strategies, timely recognition, and improved therapies.

**Funding:**

Netherlands Organization for Health Research and Development (ZonMW).

**Research in context**

**Evidence before this study**

We searched PubMed up to February 2025 for studies on outcome and neurological sequelae following bacterial meningitis caused by various pathogens. Search terms included: “bacterial meningitis”, “outcome”, “neurological sequelae”, “*Streptococcus pneumoniae*”, “*Neisseria meningitidis*”, “*Listeria monocytogenes*”, “*Haemophilus influenzae*”, and “*Streptococcus pyogenes*”. Various observational studies described the clinical characteristics, complications, and outcome of bacterial meningitis separately for different causative pathogens. A cohort study published in 2016 highlighted risk factors for adverse outcome in adult bacterial meningitis, but data on neurological sequalae following bacterial meningitis and detailed pathogen-specific outcomes remain limited.

**Added value of this study**

This study provides a pathogen-specific analysis of outcome in 2974 adult patients with bacterial meningitis from a nationwide cohort over 18 years. We identify risk factors for unfavourable outcome and neurological sequelae, and we compare outcomes across the most common causative pathogens. By including all episodes, we provide a comprehensive overview of clinical course, highlight differences between pathogens, and offer novel insights into prognosis and disease burden.

**Implications of all the available evidence**

Our findings underscore the persistent burden of bacterial meningitis in adults: one in six patients dies and half of survivors experience neurological sequelae – rates that have not improved over the past 18 years, despite a 90% adherence rate to adjunctive dexamethasone treatment. Pathogen-specific differences were evident, with *L*. *monocytogenes*, *S*. *pyogenes*, and *S*. *pneumoniae* associated with the highest fatality rates. These data reinforce the urgent need for improved prevention and treatment, including enhanced vaccine strategies, timely recognition and randomized controlled trials of new adjunctive therapies to decrease central nervous system inflammation.

## Introduction

Bacterial meningitis is associated with high mortality and morbidity rates,^1^ despite the widespread implementation of vaccination, effective antibiotics, and introduction of adjunctive dexamethasone over the last two decades.^2^^,^^3^ Common complications include hearing loss,^4^ cognitive impairment,^5^ hydrocephalus,^6^ seizures,^7^ and cerebrovascular events.^8^ Consequently, many patients suffer unfavourable functional outcomes with a substantial proportion requiring rehabilitation and long-term care. Previous studies have primarily focussed on individual pathogens or complications, involved relatively small sample sizes, and often lacked detailed information on neurological sequelae and pathogen-specific differences. As a result, the overall burden and determinants of functional outcomes across different causative pathogens remain insufficiently defined. A better understanding of the incidence and risk factors for these outcomes could inform improved screening and rehabilitation strategies, strengthen prevention and vaccination programmes, and guide the development of new targeted therapies. This study provides a comprehensive analysis of bacterial meningitis outcomes in adults, focusing on pathogen-specific differences, risk factors for unfavourable outcomes, and neurological sequelae. The size of our dataset allows for in-depth comparisons between pathogens and provide novel insights into the impact of bacterial meningitis.

## Methods

### Study population

We conducted a nationwide prospective cohort study in the Netherlands (MeninGene) from January 2006 to January 2024, including patients aged 16 years or older diagnosed with community-acquired bacterial meningitis. Bacterial meningitis was defined as a positive cerebrospinal fluid (CSF) culture, or a combination of a positive blood culture, positive bacterial polymerase chain reaction (PCR), or positive bacterial antigen test in the CSF with at least one individual predictive CSF finding for bacterial meningitis, defined as a leukocyte count of >2000 cells/mm^3^, a polymorphonuclear leukocyte count of >1180 cells/mm^3^, a glucose level of <1·9 mmol/L, a ratio of CSF glucose to blood glucose of <0·23, or a protein level of >2·2 g/L.^9^

Patients were identified through the database of the Netherlands Reference Laboratory for Bacterial Meningitis (NRLBM), which receives approximately 90% of the isolates of all adult patients with bacterial meningitis.^10^ The NRLBM provided daily updates on the hospitals where these patients were admitted, allowing for subsequent contact with the treating physicians. Physicians could also include patients without notification by the NRLBM. Written informed consent was obtained from all patients or their legally authorized representatives. We excluded episodes of hospital-acquired bacterial meningitis, defined as bacterial meningitis that occurred during hospitalization or within one week after discharge. Patients were also excluded if they had experienced significant head trauma or undergone neurosurgery in the previous month, or had an implanted neurosurgical device. Recurrent episodes of bacterial meningitis were excluded if previously included in the cohort.

### Procedures

Patient sex was recorded as male or female by the treating physician, according to the clinical record. Patients were classified as immunocompromised if they were receiving immunosuppressive drugs, were asplenic, had a recent history of alcohol use disorder, had diabetes mellitus, active cancer, or an infection with human immunodeficiency virus (HIV) with a CD4 count below 500 cells/μL. Neurological examination was performed at least upon admission and discharge by the treating physician, typically a neurologist. Data on patients’ history, symptoms and signs at admission, laboratory findings at admission, clinical course, outcome, neurological findings at discharge, and treatment were prospectively collected by the treating physician using online case record forms. Systemic complications were defined as the presence of circulatory shock, respiratory failure, hyponatremia (blood sodium level <130 mmol/L), persistent fever (lasting ≥10 days), pneumonia, or arthritis. Neurological complications were defined by the occurrence of seizures, focal neurological deficits, brain abscess, generalized cerebral oedema, hydrocephalus, subdural empyema, cerebral infarction, cerebral haemorrhage, or cerebral venous sinus thrombosis. Cerebral infarction was clinically diagnosed by the treating physician based on a combination of clinical presentation and radiological imaging. The Glasgow Outcome Scale (GOS) was used to classify outcomes upon hospital discharge, with unfavourable outcomes defined as GOS scores between one and four (Supplementary Table 1).^11^ Cognitive impairment was clinically diagnosed at discharge by the treating physician and was not routinely confirmed through neuropsychological testing.

### Statistical analysis

We determined the incidence by calculating the number of new episodes per epidemiological year (July to June) per 100,000 adults (>16 years) in the Netherlands, based on the population as of January within that same epidemiological year. To analyse potential changes in incidence, incidence rate ratios (IRRs) with 95% confidence intervals (CIs) were calculated using the Epitools package (version 0 5–10 1) in RStudio.

Categorical variables were presented as counts with percentages, while continuous variables were expressed as medians with interquartile ranges (IQR). Proportions of categorical variables were compared using Fisher's exact test. Differences in continuous variables were assessed using either t-test, or the Mann–Whitney U test for non-normally distributed data. Confidence intervals of proportions were calculated using the Wilson score method. Logistic regression analysis was performed to assess whether clinical outcomes (GOS score 1–4 vs. GOS score 5) changed over the study period, with year of admission as the predictor variable. Linearity between admission year and the logit of clinical outcome was assessed and confirmed using the Box–Tidwell test.^12^ Logistic regression was also used to evaluate the association between potential predictors and outcomes. We selected potential predictors of unfavourable outcome based on prior research, and clinical and pathophysiological relevance. The linearity of the association between continuous predictors and the logit of clinical outcome was assessed by visual inspection. When no linear relationship was found, the continuous variable was categorized for further analysis. Odds ratios (ORs) were reported with 95% CIs. Overall p-values for categorical variables with more than two categories were obtained by performing a Likelihood Ratio Test (LRT) on the multivariable logistic regression model.

Multiple imputation was applied to handle missing data in the multivariable analysis. We used all predictors included in the model to impute missing values with the Mice package (version 3.16) in RStudio, combining the results of 50 imputation sets with 10 iterations per set to derive the final estimates for the multivariable model. Passive imputation was used for the imputation of combined variables, ensuring that the values were estimated based on existing data patterns.^13^ Kaplan–Meier survival curves were generated to conduct survival analyses, and differences in survival distributions were assessed using the log-rank test. Pairwise comparisons were performed using the log-rank test with Bonferroni correction to adjust for multiple comparisons. Statistical tests were two-tailed and p-values below 0·05 were considered statistically significant. Imputation and all statistical analyses were conducted in R version 4.4.2. The study adhered to the standards for the Strengthening the Reporting of Observational studies in Epidemiology (STROBE) checklist.

### Study approval

The MeninGene study was approved by the Medical Ethics Committee of the Amsterdam UMC, location AMC, Amsterdam, The Netherlands (number METC 2013_043). Written informed consent was obtained from all patients or their legally authorized representatives.

### Role of funding source

The funding source had no role in the design of the study, data collection, data analysis, data interpretation, writing of the manuscript, or the decision to submit for publication.

## Results

**Fig. 1:**
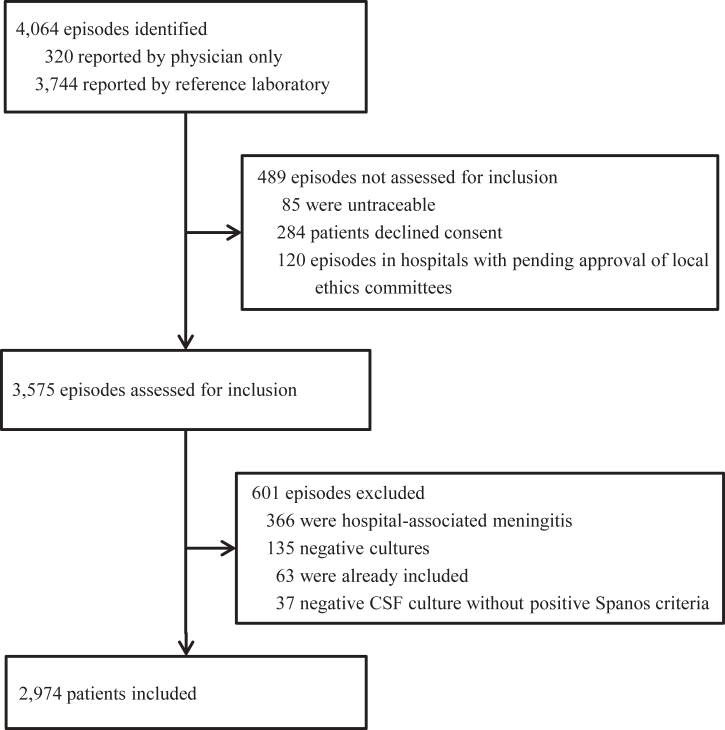
Selection of patients. CSF = cerebrospinal fluid. Spanos criteria: glucose level of <1·9 mmol/L, a ratio of CSF glucose to blood glucose of <0·23, a protein level of >220 mg/dL, a leukocyte count of >2000 cells/mm^3^, or a polymorphonuclear leukocyte count of >1180 cells/mm^3^.

**Fig. 2:**
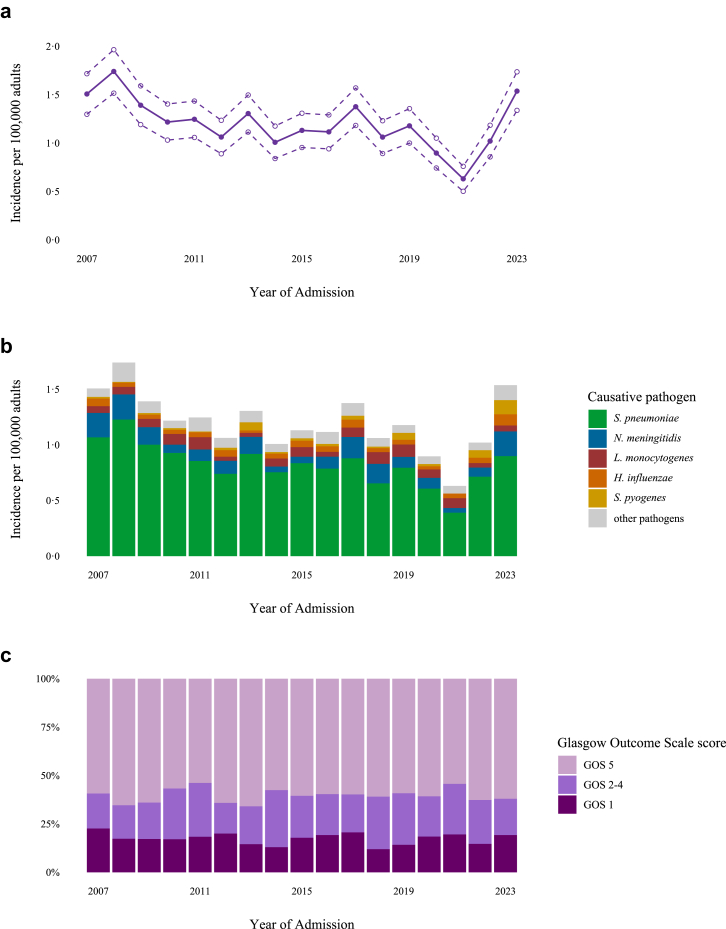
Incidence and annual clinical outcome of community-acquired bacterial meningitis in adults in the Netherlands. (a) The incidence rate with 95% confidence interval (CI) of all included episodes of bacterial meningitis per 100,000 adults per year, (b) and incidence rates per causative pathogen. Incidence was calculated as the number of new episodes per epidemiological year (July 1–June 30) per 100,000 adult patients (>16 years old on Jan 1). (c) Annual clinical outcome of bacterial meningitis, presented as percentages of patients with a favourable, unfavourable and fatal outcome. GOS = Glasgow Outcome Scale, GOS 1 = death, GOS 2 = vegetative state, GOS 3 = severe disability, GOS 4 = moderate disability, GOS 5 = mild or no disability.

A total of 2974 patients were included (Fig. 1). *S*. *pneumoniae* (2029 patients; 68%) was the leading cause, followed by *N*. *meningitidis* (329 patients; 11%), *L. monocytogenes* (182 patients; 6%), *H*. *influenzae* (119 patients; 4%) and *S*. *pyogenes* (83 patients; 3%). In 2020–2021, during the COVID-19 pandemic, incidence declined to 0·63 episodes per 100,000 adults per year, but rebounded rapidly to 1·54 in 2022–2023 after the COVID-19 related restrictions were lifted (Fig. 2a). This temporary change was driven by reduction in meningitis caused by *S*. *pneumoniae* and *N*. *meningitidis* in the period 2020–2021 (compared to the relatively stable reference period 2014–2018; IRR 0·50 [95% CI 0·38–0·66] and IRR 0·35 [95% CI 0·15-0·80], respectively; Fig. 2b), and increase in disease caused by *N*. *meningitidis*, *H*. *influenzae* and *S*. *pyogenes* in the period 2022–2023 (compared to 2014–2018; IRR 1·89 [1·26–2·83], IRR 1·96 [1·07–3·57] and IRR 5·95 [3·02–11·70], respectively). The incidence of *L*. *monocytogenes* remained stable during this period (2020–2021 compared to 2014–2018; IRR 1·13 [0·62–2·08] and 2022–2023 compared to 2014–2018; IRR 0·68 [0·33–1·43]).

The median patient age was 61 years (IQR 48–70) with an equal proportion of males and females (1480 of 2974 [50%] were female; Table 1). An immunocompromised state was noted in 912 of 2973 patients (31%), with diabetes mellitus, alcohol use disorder, and malignancy as most common risk factors. Of 2971 patients, 125 (4%) had a history of community-acquired bacterial meningitis. Extra-meningeal infection foci were identified in 1372 of 2950 patients (47%), most common otitis, sinusitis, pneumonia, and endocarditis. Common symptoms and signs were headache (2017 of 2529 [80%]), fever (2072 of 2889 [72%]), neck stiffness (1952 of 2703 [72%]), and altered mental status (2087 of 2958 [71%]), but the classic triad of fever, neck stiffness, and altered mental status was present in only 1081 of 2795 patients (39%). Cranial nerve palsies were present in 202 of 2477 patients (8%), and other focal neurological signs in 618 of 2675 patients (23%).

**Table 1:** Clinical characteristics of 2974 patients with community-acquired bacterial meningitis and of the most common causative pathogens.

Characteristic	All patients^a^ N = 2974	*S*. *pneumoniae* N = 2029	*N*. *meningitidis* N = 329	*L*. *monocytogenes* N = 182	*H*. *influenzae* N = 119	*S*. *pyogenes* N = 83
Age–years	61 (48–70)	62 (52–70)	26 (18–54)	70 (61–78)	61 (47–72)	58 (44–66)
Female sex	1480/2974 (50%)	1017/2029 (50%)	176/329 (53%)	66/182 (36%)	75/119 (63%)	51/83 (61%)
**Predisposing conditions**						
Immunocompromised before admission	912/2973 (31%)	628/2028 (31%)	43/329 (13%)	121/182 (66%)	26/119 (22%)	15/83 (18%)
Diabetes mellitus	395/2957 (13%)	291/2015 (14%)	21/327 (6%)	31/182 (17%)	11/118 (9%)	10/83 (12%)
Immunosuppressive drug use	291/2955 (10%)	149/2013 (7%)	12/328 (4%)	87/182 (48%)	12/118 (10%)	4/83 (5%)
Active cancer	172/2958 (6%)	114/2018 (6%)	1/328 (0%)	32/181 (18%)	5/118 (4%)	2/83 (2%)
History of splenectomy	53/2968 (2%)	51/2024 (3%)	0/329 (0%)	1/182 (1%)	0/119 (0%)	0/83 (0%)
Alcoholism	188/2955 (6%)	141/2017 (7%)	9/328 (3%)	14/180 (8%)	1/117 (1%)	1/82 (1%)
Known HIV^b^	16/2963 (1%)	12/2023 (1%)	3/329 (1%)	1/179 (1%)	0/119 (0%)	0/83 (0%)
Otitis	869/2858 (30%)	736/1945 (38%)	9/315 (3%)	2/174 (1%)	42/113 (37%)	68/83 (82%)
Sinusitis	374/2832 (13%)	293/1920 (15%)	11/313 (4%)	5/175 (3%)	24/114 (21%)	18/83 (22%)
Pneumonia	291/2873 (10%)	237/1950 (12%)	7/324 (2%)	17/175 (10%)	9/114 (8%)	5/83 (6%)
Endocarditis	66/2862 (2%)	30/1945 (2%)	0/324 (0%)	0/175 (0%)	0/113 (0%)	0/83 (0%)
CSF leak	72/2940 (2%)	51/2004 (3%)	1/324 (0%)	0/182 (0%)	12/117 (10%)	0/83 (0%)
Recurrent CABM	125/2971 (4%)	92/2026 (5%)	9/329 (3%)	0/182 (0%)	16/119 (13%)	1/83 (1%)
**Symptoms on presentation**						
Headache	2017/2529 (80%)	1335/1685 (79%)	277/304 (91%)	110/154 (71%)	94/110 (85%)	54/71 (76%)
Fever on admission	2072/2889 (72%)	1460/1969 (74%)	165/321 (51%)	147/175 (84%)	80/114 (70%)	63/82 (77%)
Neck stiffness	1952/2703 (72%)	1351/1836 (74%)	229/302 (76%)	94/157 (60%)	74/113 (65%)	54/77 (70%)
Triad^c^	1081/2795 (39%)	852/1885 (45%)	62/320 (19%)	55/171 (32%)	27/115 (23%)	24/80 (30%)
GCS score on admission	11 (9–14)	10 (8–13)	14 (11–15)	13 (10–15)	14 (11–15)	13 (10–14)
Altered mental status (<14)	2087/2958 (71%)	1612/2020 (80%)	153/327 (47%)	99/179 (55%)	51/118 (43%)	50/82 (61%)
Coma (<8)	620/2958 (21%)	509/2020 (25%)	33/327 (10%)	19/179 (11%)	13/118 (11%)	12/82 (15%)
Cranial nerve palsy	202/2477 (8%)	148/1666 (9%)	6/288 (2%)	15/153 (10%)	8/110 (7%)	9/65 (14%)
Seizures	244/2857 (9%)	202/1939 (10%)	11/320 (3%)	8/174 (5%)	4/116 (3%)	6/82 (7%)
Focal neurologic signs on admission	618/2675 (23%)	459/1782 (26%)	34/317 (11%)	39/170 (23%)	18/113 (16%)	22/79 (28%)
Hemiparesis	160/2594 (6%)	122/1726 (7%)	7/316 (2%)	6/163 (4%)	4/109 (4%)	8/76 (11%)
Aphasia	433/2844 (15%)	321/1931 (17%)	26/316 (8%)	31/172 (18%)	14/115 (12%)	16/80 (20%)
**Laboratory findings**						
CRP in blood–mg/L	192 (88–301)	202 (97–315)	222 (140–303)	86 (40–171)	119 (50–220)	244 (166–332)
CSF Leukocytes–cells/mm^3^	2400 (587–6891)	2339 (511–6638)	6112 (2230–12,375)	812 (356–1749)	3930 (2000–8208)	1641 (506–4764)
CSF granulocytes proportion–%	94 (85–98)	95 (87–98)	95 (90–98)	82 (61–91)	89 (80–95)	92 (84–96)
CSF protein–g/L	3·90 (2·30–6·10)	4·26 (2·55–6·37)	3·93 (2·05–6·19)	2·55 (1·74–3·47)	3·21 (1·93–5·95)	3·50 (1·68–6·07)
CSF glucose–mmol/L	0·50 (0·10–2·50)	0·20 (0·10–2·00)	0·60 (0·10–2·80)	2·05 (0·90–3·20)	1·00 (0·20–2·45)	1·90 (0·15–3·90)

Data are shown as median [IQR] or n/N (%).

HIV = human immunodeficiency virus, CABM = community-acquired bacterial meningitis, GCS = Glasgow Coma Scale, CSF = cerebrospinal fluid, CRP = C-reactive protein.

a *Streptococcus pneumoniae* (n = 2029), *Neisseria meningitidis* (n = 329), *Listeria monocytogenes* (n = 182), *Haemophilus influenzae* (n = 119), *Streptococcus pyogenes* (n = 83), *Staphylococcus aureus* (n = 51), *Streptococcus agalactiae* (n = 41), oral streptococci (n = 32), *Escherichia coli* (n = 24), *Streptococcus anginosus* group (n = 19), *Klebsiella pneumoniae* (n = 11), *Streptococcus suis* (n = 7), *Streptococcus bovis*/*Streptococcus equinus* complex (n = 7), *Capnocytophaga canimorsus* (n = 6), *Streptococcus dysgalactiae* (n = 5), *Pseudomonas aeruginosa* (n = 4), *Streptococcus equi* (n = 3), *Enterococcus faecalis* (n = 2), *Campylobacter fetus* (n = 2), *Haemophilus parainfluenzae* (n = 2), *Streptococcus thermophilus* (n = 2), *Nocardia farcinica* (n = 1), *Salmonella enterica* (n = 1), *Rhizobium radiobacter* (n = 1), *Aggregatibacter aphrophilus* (n = 1), *Fusobacterium necrophorum* (n = 1), *Moraxella osloensis* (n = 1), *Shewanella algae* (n = 1), Streptococcus not specified (n = 6).

b Immunocompromised status due to HIV was defined as CD4 <500 cells/μL.

c Triad of fever, neck stiffness and altered mental status.

CSF analysis revealed a median leukocyte count of 2400 cells/mm^3^ (IQR 587–6891), a median protein concentration of 3·90 g/L (IQR 2·30–6·10), and a median glucose level of 0·50 mmol/L (IQR 0·10–2·50). CSF cultures were positive in 2667 of 2974 patients (90%), while blood cultures yielded positive results in 1964 of 2572 patients (76%). Initial antibiotic therapy consisted of a combination of amoxicillin and a third-generation cephalosporin in 1599 of 2866 patients (56%). Monotherapy with a third-generation cephalosporin was administered to 692 of 2866 patients (24%), and penicillin or amoxicillin alone was used in 394 of 2866 patients (14%). Adjunctive dexamethasone was given to 2600 of 2897 patients (90%), with 2437 of these 2600 (94%) receiving 10 mg intravenously every 6 h for 4 days, initiated before or with the first dose of parenteral antibiotics.

The classic triad of fever, neck stiffness, and altered mental status was most common in pneumococcal meningitis (852/1885 [45%] vs. 229/910 [25%] in all other patients, p < 0·0001; Table 1). Patients with meningococcal meningitis were younger (26 [IQR 18–54] vs. 62 [IQR 52–71] years, p < 0·0001), less likely to have underlying immunocompromising conditions (43/329 [13%] vs. 869/2644 [33%], p < 0·0001) or extra-meningeal foci of infection (26/327 [8%] vs. 1346/2623 [51%], p < 0·0001). Patients with listeria meningitis were more likely to be immunocompromised (121/182 [66%] vs. 791/2791 [28%], p < 0·0001), with a high proportion using immunosuppressive drugs (87/182 [48%] vs. 204/2773 [7%], p < 0·0001). They also had lower median CSF leukocyte counts (812 [IQR 356–1749] vs. 2731 [IQR 626–7331] cells/mm^3^, p < 0·0001). In 35 of 161 patients (22%) with listeria meningitis, amoxicillin was not administrated on the first day of admission. Patients with *S*. *pyogenes* meningitis were more likely to have an extra-meningeal focus of infection (74/83 [89%] vs. 1298/2867 [45%], p < 0·0001; otitis in 68 of these 83 patients [82%]).

During clinical course, systemic complications occurred in 1309 of 2791 patients (47%), consisting of circulatory shock in 361 (13%), respiratory failure in 723 (25%), hyponatremia in 297 (11%), persistent fever in 322 (12%), pneumonia in 437 (16%), and arthritis in 90 patients (3%; Table 2). Neurological complications were observed during clinical course in 1376 of 2792 patients (49%), including focal neurological deficits in 701 (25%), seizures in 535 (18%), cerebral infarction in 279 (10%), hydrocephalus in 206 (7%), subdural empyema in 64 (2%), and brain abscess in 60 patients (2%). Patients with *S*. *pyogenes* meningitis had high rates of subdural empyema, occurring in 17 of 81 cases (21%) compared to 47 of 2601 (2%) in other patients (p < 0·0001).

**Table 2:** Complications and outcome of 2974 patients with community-acquired bacterial meningitis and of the most common causative pathogens.

Characteristic	All patients^a^ N = 2974	*S*. *pneumoniae* N = 2029	*N*. *meningitidis* N = 329	*L*. *monocytogenes* N = 182	*H*. *influenzae* N = 119	*S*. *pyogenes* N = 83
**Complications during admission**						
Systemic complications	1309/2791 (47%)	957/1901 (50%)	77/302 (25%)	101/176 (57%)	25/114 (22%)	47/82 (57%)
Circulatory shock	361/2827 (13%)	243/1922 (13%)	30/318 (9%)	25/171 (15%)	3/112 (3%)	23/83 (28%)
Respiratory failure	723/2869 (25%)	557/1953 (29%)	26/323 (8%)	50/176 (28%)	9/114 (8%)	26/81 (32%)
Hyponatremia	297/2810 (11%)	196/1914 (10%)	18/314 (6%)	35/169 (21%)	11/115 (10%)	11/81 (14%)
Persistent fever	322/2798 (12%)	236/1897 (12%)	6/321 (2%)	28/173 (16%)	5/114 (4%)	16/79 (20%)
Pneumonia	437/2801 (16%)	357/1901 (19%)	9/314 (3%)	28/172 (16%)	10/115 (9%)	8/81 (10%)
Arthritis	90/2818 (3%)	64/1913 (3%)	12/318 (4%)	1/172 (1%)	3/114 (3%)	1/82 (1%)
Neurological complications	1376/2792 (49%)	1036/1917 (54%)	66/310 (21%)	77/163 (47%)	29/107 (27%)	57/80 (71%)
Seizures	535/2971 (18%)	433/2027 (21%)	16/329 (5%)	31/182 (17%)	10/119 (8%)	25/83 (30%)
Neurological deficits	701/2761 (25%)	509/1874 (27%)	41/317 (13%)	34/165 (21%)	11/113 (10%)	41/79 (52%)
Cerebral infarction	279/2788 (10%)	224/1900 (12%)	6/316 (2%)	6/167 (4%)	2/113 (2%)	13/78 (17%)
Cerebral haemorrhage	55/2788 (2%)	38/1900 (2%)	0/316 (0%)	4/167 (2%)	2/113 (2%)	3/78 (4%)
Cerebral abscess	60/2686 (2%)	28/1907 (1%)	3/234 (1%)	8/156 (5%)	0/103 (0%)	3/81 (4%)
Subdural empyema	64/2682 (2%)	44/1902 (2%)	1/234 (0%)	0/157 (0%)	0/103 (0%)	17/81 (21%)
Hydrocephalus	206/2791 (7%)	149/1908 (8%)	5/313 (2%)	19/166 (11%)	4/111 (4%)	8/78 (10%)
Generalized cerebral oedema	290/2683 (11%)	230/1905 (12%)	17/234 (7%)	5/156 (3%)	5/102 (5%)	15/81 (19%)
Cerebral venous thrombosis	62/2748 (2%)	48/1870 (3%)	3/311 (1%)	0/167 (0%)	1/111 (1%)	7/75 (9%)
**Outcome**						
Glasgow Outcome Scale						
1 (death)	516/2974 (17%)	365/2029 (18%)	11/329 (3%)	58/182 (32%)	4/119 (3%)	16/83 (19%)
2 (vegetative state)	3/2974 (0%)	2/2029 (0%)	0/329 (0%)	0/182 (0%)	0/119 (0%)	0/83 (0%)
3 (severe disability)	138/2974 (5%)	105/2029 (5%)	5/329 (2%)	8/182 (4%)	1/119 (1%)	6/83 (7%)
4 (moderate disability)	504/2974 (17%)	373/2029 (18%)	30/329 (9%)	34/182 (19%)	16/119 (13%)	15/83 (18%)
5 (mild or no disability)	1813/2974 (61%)	1184/2029 (58%)	283/329 (86%)	82/182 (45%)	98/119 (82%)	46/83 (55%)

Data are shown as median [IQR] or n/N (%).

a *Streptococcus pneumoniae* (n = 2029), *Neisseria meningitidis* (n = 329), *Listeria monocytogenes* (n = 182), *Haemophilus influenzae* (n = 119), *Streptococcus pyogenes* (n = 83), *Staphylococcus aureus* (n = 51), *Streptococcus agalactiae* (n = 41), oral streptococci (n = 32), *Escherichia coli* (n = 24), *Streptococcus anginosus* group (n = 19), *Klebsiella pneumoniae* (n = 11), *Streptococcus suis* (n = 7), *Streptococcus bovis*/*Streptococcus equinus* complex (n = 7), *Capnocytophaga canimorsus* (n = 6), *Streptococcus dysgalactiae* (n = 5), *Pseudomonas aeruginosa* (n = 4), *Streptococcus equi* (n = 3), *Enterococcus faecalis* (n = 2), *Campylobacter fetus* (n = 2), *Haemophilus parainfluenzae* (n = 2), *Streptococcus thermophilus* (n = 2), *Nocardia farcinica* (n = 1), *Salmonella enterica* (n = 1), *Rhizobium radiobacter* (n = 1), *Aggregatibacter aphrophilus* (n = 1), *Fusobacterium necrophorum* (n = 1), *Moraxella osloensis* (n = 1), *Shewanella algae* (n = 1), Streptococcus not specified (n = 6).

**Fig. 3:**
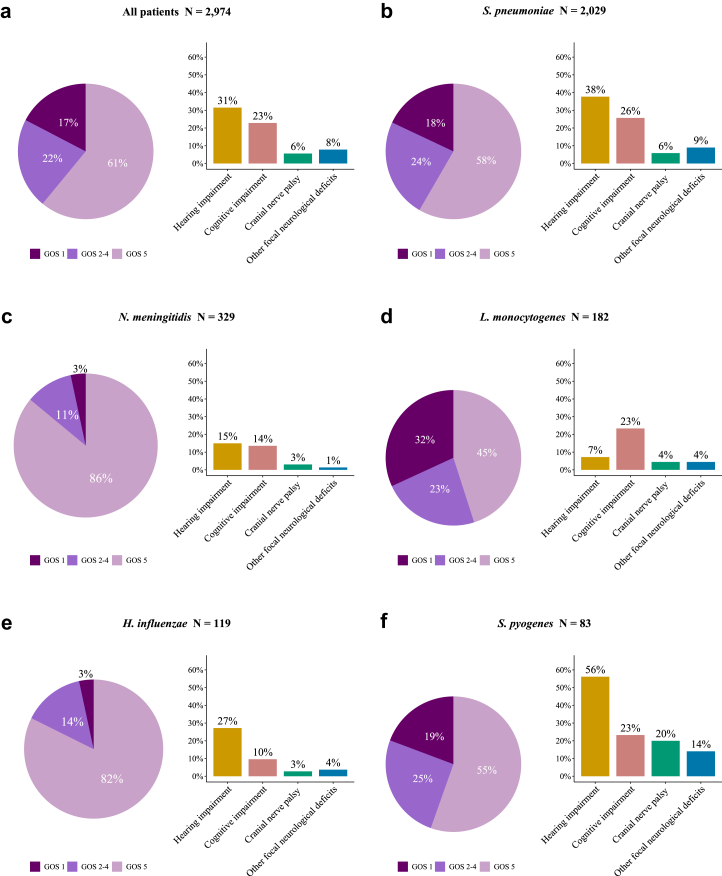
Clinical outcome and neurological sequelae following bacterial meningitis. (a) Reported clinical outcome (left) and neurological deficits at discharge (right) of all community-acquired bacterial meningitis patients in the Netherlands, (b–f) and of the most common causative pathogens. GOS = Glasgow Outcome Scale, GOS 1 = death, GOS 2 = vegetative state, GOS 3 = severe disability, GOS 4 = moderate disability, GOS 5 = mild or no disability.

**Fig. 4:**
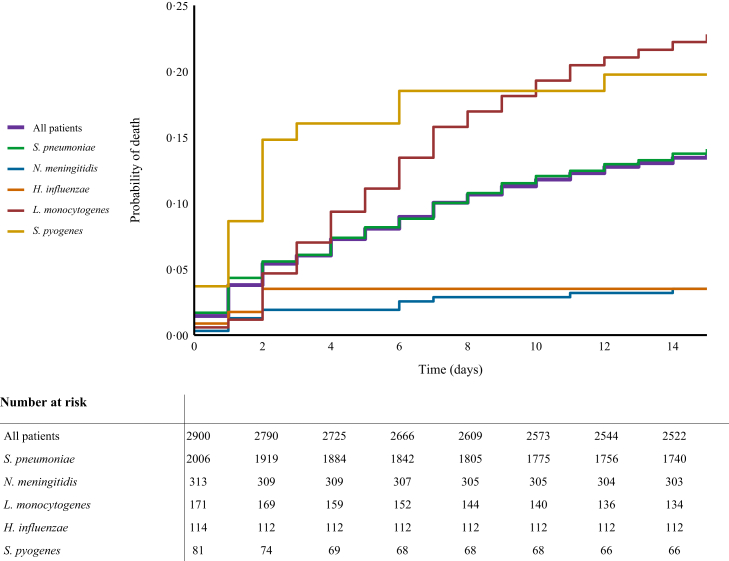
Probability of death in patients with bacterial meningitis. Kaplan–Meier survival curve illustrating the probability of death for all patients diagnosed with bacterial meningitis (purple) and the probability of death stratified by the most common causative pathogens (other colours). Patients discharged within 14 days of admission were considered alive until day 14 and were not censored before that time.

Unfavourable outcome was reported in 1161 of 2974 patients (39%; 95% CI 37–41) and 516 of 2974 (17%; 95% CI 16–19) died (Fig. 3a). The clinical outcome remained stable throughout the study period. Trend analysis yielded an odds ratio of 1·00 (95% CI 0·99–1·02) for unfavourable outcome per year of admission (β = 0·004, where β represents the log OR), and an odds ratio of 0·99 (95% CI, 0·97–1·01) for death (β = −0·007; Fig. 2c). The clinical outcomes varied per pathogen (Fig. 3b–f): the rate of unfavourable outcome was 42% (845/2029; 95% CI 40–44) for *S*. *pneumoniae*, 14% (46/329; 95% CI 11–18) for *N*. *meningitidis*, 55% (100/182; 95% CI 48–62) for *L*. *monocytogenes*, 18% (21/119; 95% CI 12–26) for *H*. *influenzae*, and 45% (37/83; 95% CI 42–53) for *S*. *pyogenes*. The highest case fatality rates were observed in *L*. *monocytogenes* (58/182 [32%; 95% CI 26–39]), *S*. *pyogenes* (16/83 [19%; 95% CI 12–29]), and *S*. *pneumoniae* (365/2029 [18%; 95% CI 16–20]). The median time to death among deceased patients was 6 days (IQR 2–14). Kaplan–Meier survival analysis showed differences in survival times across pathogens (χ^2^ = 65·5, df = 4, p < 0·001; Fig. 4); deceased patients with *S*. *pyogenes* meningitis had a median time to death of 2 days (IQR 1–2·25), while those with *L*. *monocytogenes* meningitis had a median time to death of 9 days (IQR 4–18).

The median time to hospital discharge among surviving patients was 15 days (IQR 11–22) and 621 of 2418 (24%) were discharged to non-home settings (422 [17%] to rehabilitation centres, and 124 [5%] to nursing homes). Neurological sequelae at discharge were observed in 1146 of 2088 surviving patients (55%), including hearing impairment in 692 of 2197 (31%), cognitive impairment in 481 of 2108 (23%), cranial nerve palsies in 124 of 2220 (6%), and other focal neurological deficits in 177 of 2261 patients (8%; Fig. 3a).

Hearing impairment and focal neurological deficits were most common among patients with *S*. *pneumoniae* and *S*. *pyogenes* meningitis (hearing impairment in 558 of 1481 *S*. *pneumoniae* patients [38%] and 32 of 57 *S*. *pyogenes* patients [56%], vs. 102 of 659 [15%] in all other patients [p < 0·0001], and focal neurological deficits in 137 of 1523 *S*. *pneumoniae* patients [9%], and nine of 64 *S*. *pyogenes* patients [14%], vs. 31 of 674 [5%] in all other patients [p = 0·00015]), while cognitive impairment was prevalent across all pathogens (Table 2; Fig. 3b–f). Cognitive impairment occurred in the absence of other neurological sequelae in 192 of 2088 surviving patients (9%).

**Fig. 5:**
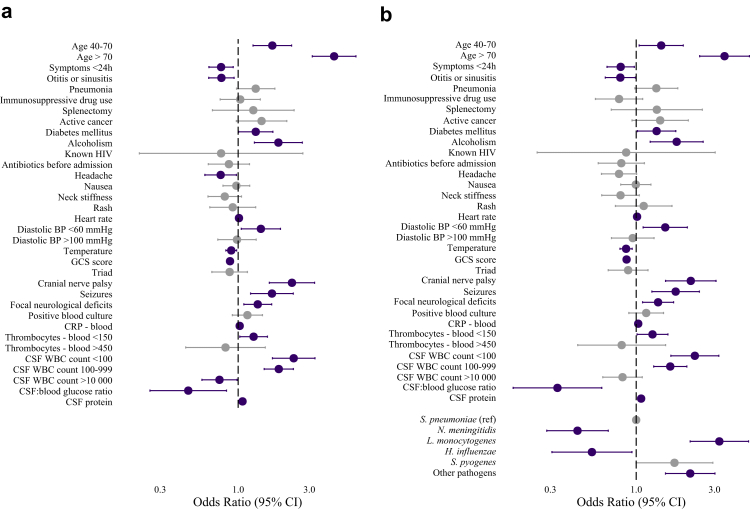
Predictors of unfavourable outcome in bacterial meningitis. (a) Forest plot depicting predictors of unfavourable outcomes in bacterial meningitis of any pathogen, (b) and adjusted for the most common causative pathogens. Odds ratios (OR) with 95% confidence intervals (CI) are plotted on a logarithmic scale. ORs for CRP are expressed per 10 mg/L increase. Significant predictors are highlighted in dark purple, non-significant predictors are shown in grey. The dashed vertical line represents OR = 1.

For variables included in the multivariable analysis, 6% of the data was missing and handled using multiple imputation. In this analysis, several factors were found to be associated with unfavourable outcome in bacterial meningitis caused by any pathogen (Table 3; Fig. 5a). Advanced age, symptoms lasting more than 24 h before hospital presentation, absence of otitis or sinusitis, diabetes mellitus, alcoholism, absence of fever, absence of headache, elevated heart rate, low diastolic blood pressure, low GCS score, cranial nerve palsy, seizures, focal neurological deficits at admission, elevated CRP levels in blood, low thrombocyte count in blood, low CSF leukocyte count, low CSF-to-blood glucose ratio, and elevated CSF protein levels were all associated with unfavourable outcome. Adjusted for all predictors, *L*. *monocytogenes* meningitis was associated with a higher risk of unfavourable outcome compared to pneumococcal meningitis (univariable OR 1·71 [95% CI 1·26–2·32], and multivariable adjusted OR (aOR) 3·21 [95% CI 2·13–4·84]; Fig. 5b). Dexamethasone treatment was associated with a decreased risk of unfavourable outcome (univariable OR 0·45 [95% CI 0.36–0.58], and multivariable aOR 0·53 [95% CI 0·40–0·72]) and death (univariable OR 0·38 [95% CI 0.29–0.50], and multivariable aOR 0·47 [95% CI 0·34–0·66]). In pneumococcal meningitis, the multivariable aOR for the association between dexamethasone treatment and unfavourable outcome and death were 0·56 (95% CI 0·38–0·83) and 0·57 (95% CI 0·37–0·90), respectively. Supplementary Table 2 presents the multivariable analysis restricted to patients with pneumococcal meningitis.

**Table 3:** Factors associated with unfavourable outcome in patients with bacterial meningitis.

	Favourable outcome N = 1813	Unfavourable outcome N = 1161	Univariable odds ratio for unfavourable outcome	Multivariable odds ratio for unfavourable outcome	p value of multivariable analysis
Age (years)	58 (42–67)	65 (56–75)			**<0·0001**^c^
16–39	398/1813 (22%)	84/1161 (7%)	*Reference*	*Reference*	
40–70	1141/1813 (63%)	648/1161 (56%)	2·69 (2·10–3·49)	1·69 (1·26–2·28)	0·00053
>70	274/1813 (15%)	429/1161 (37%)	7·42 (5·63–9·86)	**4·40 (3·14**–**6·16)**	**<0·0001**
Symptoms <24h	875/1768 (49%)	471/1086 (43%)	0·78 (0·67–0·91)	**0·77 (0·64**–**0·93)**	**0·0060**
Otitis or sinusitis	745/1776 (42%)	344/1104 (31%)	0·63 (0·53–0·73)	**0·77 (0·64**–**0·94)**	**0·010**
Pneumonia	121/1775 (7%)	170/1098 (15%)	2·50 (1·96–3·21)	1·31 (0·98–1·76)	0·073
Immunosuppressive drug use	159/1807 (9%)	132/1148 (11%)	1·35 (1·05–1·72)	1·03 (0·76–1·41)	0·84
Splenectomy	26/1809 (1%)	27/1159 (2%)	1·64 (0·95–2·83)	1·26 (0·67–2·38)	0·47
Active cancer	68/1805 (4%)	104/1153 (9%)	2·53 (1·85–3·48)	1·44 (0·97–2·12)	0·069
Diabetes mellitus	200/1805 (11%)	195/1152 (17%)	1·64 (1·32–2·02)	**1·31 (1·01**–**1·71)**	**0·045**
Alcoholism	73/1804 (4%)	115/1151 (10%)	2·63 (1·95–3·58)	**1·86 (1·29**–**2·69)**	**0·0010**
Known HIV	10/1807 (1%)	6/1156 (1%)	0·94 (0·32–2·53)	0·77 (0·22–2·72)	0·68
Antibiotics before admission	182/1770 (10%)	93/1130 (8%)	0·78 (0·60–1·01)	0·87 (0·63–1·19)	0·38
Headache	1411/1662 (85%)	606/867 (70%)	0·41 (0·34–0·50)	**0·76 (0·60**–**0·97)**	**0·030**
Nausea	964/1570 (61%)	437/861 (51%)	0·65 (0·55–0·77)	0·97 (0·79–1·20)	0·79
Neck stiffness	1255/1677 (75%)	697/1026 (68%)	0·71 (0·60–0·85)	0·81 (0·63–1·05)	0·12
Rash	178/1607 (11%)	65/992 (7%)	0·56 (0·42–0·75)	0·92 (0·65–1·31)	0·64
Heart rate (b/min)	95 (81–109)	103 (89–120)	1·02 (1·02–1·02)	**1·01 (1·01**–**1·02)**	**<0·0001**
Diastolic BP (mmHg)	79 (69–90)	80 (70–93)			0·072^c^
<60	153/1758 (9%)	124/1106 (11%)	1·41 (1·10–1·81)	1·42 (1·05–1·92)	0·023
60–100	1460/1758 (83%)	839/1106 (76%)	*Reference*	*Reference*	
>100	145/1758 (8%)	143/1106 (13%)	1·72 (1·34–2·20)	0·98 (0·73–1·32)	0·91
Temperature (°C)	38·90 (37·90–39·60)	38·70 (37·60–39·50)	0·88 (0·83–0·93)	**0·90 (0·83**–**0·98)**	**0·013**
GCS score	12 (10–14)	10 (8–13)	0·84 (0·82–0·86)	**0·88 (0·85**–**0·91)**	**<0·0001**
Triad^a^	663/1721 (39%)	418/1074 (39%)	1·02 (0·87–1·19)	0·88 (0·67–1·15)	0·35
Cranial nerve palsy	85/1567 (5%)	117/910 (13%)	2·57 (1·92–3·45)	**2·30 (1·62**–**3·26)**	**<0·0001**
Seizures	102/1773 (6%)	142/1084 (13%)	2·47 (1·89–3·23)	**1·69 (1·21**–**2·34)**	**0·0019**
Focal neurological deficits	324/1703 (19%)	294/972 (30%)	1·85 (1·54–2·22)	**1·35 (1·09**–**1·68)**	**0·0059**
CRP–blood (mg/L)^b^	161 (74–261)	240 (127–350)	1·04 (1·03–1·04)	**1·02 (1·02**–**1·03)**	**<0·0001**
Thrombocyte count	207 (160–261)	185 (132–250)			0·064^c^
<150	365/1736 (21%)	360/1081 (33%)	1·89 (1·59–2·24)	1·27 (1·03–1·57)	0·029
150–450	1330/1736 (77%)	695/1081 (64%)	*Reference*	*Reference*	
>450	41/1736 (2%)	26/1081 (2%)	1·21 (0·73–1·99)	0·82 (0·45–1·52)	0·53
CSF WBC count (cells/mm^3^)	3224 (1067–7880)	1341 (245–5210)			<0·0001^c^
<100	105/1780 (6%)	191/1120 (17%)	4·01 (3·09-5·22)	2·36 (1·70–3·27)	<0·0001
100–999	323/1780 (18%)	324/1120 (29%)	2·18 (1·81-2·64)	1·87 (1·49–2·35)	<0·0001
1000–9999	1022/1780 (57%)	470/1120 (42%)	*Reference*	*Reference*	
>10,000	330/1780 (19%)	135/1120 (12%)	0·89 (0·71-1·12)	**0·75 (0·57**–**0·98)**	**0·037**
CSF:blood glucose ratio	0·08 (0·01–0·30)	0·02 (0·01–0·16)	0·19 (0·11–0·30)	**0·46 (0·26**–**0·84)**	**0·011**
CSF protein (g/L)	3·56 (2·14–5·80)	4·54 (2·71–6·75)	1·11 (1·08–1·13)	**1·07 (1·04**–**1·10)**	**<0·0001**
Positive blood culture	1155/1575 (73%)	809/997 (81%)	1·56 (1·29–1·90)	1·15 (0·91–1·46)	0·23

Data are shown as median [IQR] or n/N (%). Odds ratios are provided with 95% CI. The multivariable analysis used an imputed dataset with 50 imputation sets with 10 iterations per set. All variables in the table were entered in the multivariable regression model simultaneously. Bold font indicates statistically significant results in the multivariable regression.

HIV = human immunodeficiency virus, GCS = Glasgow Coma Scale, CSF = cerebrospinal fluid, WBC = white blood cell count, CRP = C-reactive protein.

a Triad of fever, neck stiffness and altered mental status.

b Odds ratios for CRP are expressed per 10 mg/L increase.

c Overall p-values for categorical variables with more than two categories were calculated using the Likelihood Ratio Test (LRT) from the multivariable logistic regression model.

## Discussion

Our findings highlight the burden of bacterial meningitis, with an annual incidence of 0·63–1·54 cases per 100,000 adults, one in six patients dying and half of survivors experiencing neurological sequelae – an outcome that has not improved over the past 18 years. Pathogen-specific differences were evident, with the causative pathogens *L*. *monocytogenes*, *S*. *pyogenes*, and *S*. *pneumoniae* linked to the highest fatality rates. Unfavourable functional outcomes were observed in a substantial proportion of patients (39%) and identified key predictors were advanced age, prolonged symptom duration before admission, markers of systemic compromise, indicators of cerebral damage, and low CSF white cell counts. Adjunctive dexamethasone therapy, introduced for adults in 2002,^14^ remains a cornerstone of treatment, contributing to improved survival and functional outcome. Nevertheless, our data show an urgent need for improved prevention and treatment, including enhanced vaccine strategies, timely recognition and randomized controlled trials of new adjunctive therapies to decrease central nervous system inflammation.

More than half of surviving patients experienced neurological impairment, most commonly hearing and cognitive deficits. Hearing loss was most prevalent in meningitis caused by *S*. *pneumoniae* and *S*. *pyogenes*. Otitis media, a known risk factor for meningitis, was present in 55% of patients with pneumococcal meningitis who developed hearing loss and may require invasive treatment.^4^ Obliteration of the cochlear lumen can occur in the acute phase of meningitis and is associated with reduced success of cochlear implantation.^15^ Early identification of hearing loss is therefore critical, and routine screening prior to hospital discharge is recommended.

Cognitive impairment was observed in approximately one-quarter of patients and was not confined to a specific causative pathogen. This finding aligns with previous studies reporting cognitive impairment following bacterial as well as viral meningitis.^5^^,^^16^^,^^17^ Cognitive impairment in this study was based on clinical assessment at discharge by the treating physician and was not routinely confirmed by neuropsychological testing, which may have led to an underestimation of its true prevalence. Detailed neuropsychological assessments have identified reduced processing speed as the primary cognitive deficit following meningitis, which correlates with lower self-reported general health and quality of life scores.^18^ Genetic variation in immune response genes may impact outcome, as illustrated by a study suggesting that inter-individual differences in the production of macrophage migration inhibitory factor following pneumococcal immune cells stimulation may contribute to long-term cognitive impairment years after pneumococcal meningitis.^19^

Focal neurological deficits were primarily attributable to underlying cerebral pathologies, including cerebrovascular events, subdural empyema, and cerebral abscesses. Cerebral infarction is a well-recognised complication of bacterial meningitis,^8^ resulting from mechanisms such as vascular endothelial injury, local vasculitis, and intravascular coagulopathy.^20^ In our cohort, cerebral infarction occurred in 12% of patients with pneumococcal meningitis and in 17% of those with *S*. *pyogenes* meningitis. These rates are lower than those reported in earlier studies conducted prior to widespread use of adjunctive dexamethasone.^21^ Patients with *S*. *pyogenes* meningitis were at particular risk for subdural empyema (21%), an important cause of epilepsy.^22^

Several clinical and laboratory parameters at presentation were independently associated with unfavourable outcome, consistent with previous research.^10^ Advanced age emerged as one of the strongest predictors, with patients over 70 years having a fourfold higher risk compared to younger adults. Notably, the case fatality rate exceeded 50% in patients aged 80 years or older. In line with prior observations, systemic comorbidities such as diabetes mellitus and alcohol use disorder were significantly associated with poor outcome, as were features of severe systemic or neurological involvement, including impaired consciousness, cranial nerve palsies, and focal neurological deficits at admission.^10^^,^^23^ Laboratory indicators of more severe disease, such as elevated inflammatory markers, low platelet counts, and abnormal cerebrospinal fluid findings (e.g., low CSF-to-blood glucose ratio, and elevated protein levels) were likewise predictive. Taken together, these findings underscore the complex interplay between host vulnerability, systemic compromise, and central nervous system injury in determining outcome. Finally, low CSF white cell counts, likely reflecting an inadequate inflammatory response allowing for higher bacterial loads, may contribute to increased mortality and morbidity.^24^

Dexamethasone was associated with favourable outcome and increased survival rates. The administration of adjunctive dexamethasone is recommended by current guidelines for patients with suspected community-acquired bacterial meningitis.^25^ The beneficial effects of dexamethasone in bacterial meningitis were first demonstrated in children,^26^ followed by a randomized controlled trial in adult patients,^14^ and further supported by a large Cochrane meta-analysis, including 4121 patients, which showed that dexamethasone significantly reduced the risk of hearing loss and neurological sequelae.^27^ Subgroup analyses revealed the greatest benefit in cases caused by *S*. *pneumoniae* and *H*. *influenzae*. Implementations studies have provided robust evidence confirming the efficacy of dexamethasone in bacterial meningitis, across all causative pathogens and in both adult and paediatric patients.^10^^,^^28^ Notably, a recent study also demonstrated a beneficial effect in patients with *L*. *monocytogenes* meningitis.^29^ Based on this evidence, a complete four-day regime of dexamethasone should be administrated in all patients with community-acquired bacterial meningitis, regardless of patient-specific risk factors, comorbidities, or suspected pathogen.

Functional outcomes among adults with bacterial meningitis remained stable over the 18-year study period of this cohort. Key predictors of poor outcome, including advanced age and dexamethasone use, also remained unchanged. Increasing awareness of the importance of early antibiotic administration would be expected to improve patient outcomes.^2^ In addition, vaccination programs in the Netherlands were expanded during the study period, with broader serotype coverage and the introduction of routine pneumococcal vaccination for older adults. The absence of measurable improvement in outcomes despite these advances further underscores the urgent need for enhanced strategies in both prevention and treatment of bacterial meningitis.

Our study has several limitations. First, we included only patients who underwent a lumbar puncture. In some cases, lumbar puncture may be delayed or deferred due to co-occurring intracerebral complications or septic shock with diffuse intravascular coagulation. The exclusion of these severely ill patients could introduce selection bias, leading to an underestimation of both the total case number and the severity of the disease. Second, patients who received antibiotics prior to lumbar puncture, often due to cranial imaging, are known to have a lower rate of CSF culture positivity. As cranial imaging is commonly performed in cases of bacterial meningitis, some cases may have been missed due to early antibiotic administration resulting in negative cultures. Third, we only collected data on neurological sequelae at discharge, lacking information on long-term outcome. Consequently, this cohort does not allow us to assess the long-term burden of bacterial meningitis. Some deficits present at discharge can improve during rehabilitation, potentially leading to better functional outcomes over time. Evidence on long-term outcomes remains limited, especially in adults. A recent study found that nearly 30% of patients exhibited neuro-functional disability 12 months after a bacterial meningitis episode, and approximately 18% of patients reported deterioration compared with hospital discharge.^30^ These findings suggest that, although some recovery is possible, a substantial proportion of patients continue to experience lasting or progressive impairment. However, this study is based on self-reported outcomes with substantial dropout, limiting its generalizability. This highlights the need for well-designed, prospective cohort studies with long-term follow-up to better understand the enduring burden of bacterial meningitis. Taken together, these data indicate that bacterial meningitis continues to have a profound impact, as reflected by the high mortality observed in our cohort and the fact that more than half of survivors were left with neurological sequelae at the time of hospital discharge.

## Contributors

ED: data curation, formal analysis, investigation, methodology, visualisation, writing – original draft. ES: investigation. NC: investigation. TvS: investigation. DK: investigation. MWB: methodology, writing – review & editing. MCB: conceptualisation, funding acquisition, methodology, project administration, resources, supervision, writing – review & editing. DvdB: conceptualisation, funding acquisition, methodology, project administration, resources, supervision, writing – review & editing.

ED, MCB and DvdB verified the data and had access to raw data. DvdB had final responsibility for decision to submit for publication.

## Data sharing statement

De-identified individual participant data underlying the findings of this study are available from the corresponding author upon reasonable request. Access to the data may be subject to institutional review and approval in accordance with patient confidentiality and ethical guidelines.

## Declaration of interests

MCB reports receiving research and innovation grants from Amsterdam UMC and the Its ME Foundation, as well as travel reimbursement from ESCMID. All other authors declare no competing interests.
