# Supplementary material for: Outcomes of adults with community-acquired bacterial meningitis in the Netherlands: a prospective nationwide cohort study
Source: Lancet Reg Health Eur. 2025 Nov 14;61:101529. doi: 10.1016/j.lanepe.2025.101529 (PMC12662076; doi:10.1016/j.lanepe.2025.101529)
Supplement: Supplementary Tables 1 and 2 [file mmc1.docx]

| **Supplementary table 1** Glasgow Outcome Scale (GOS) | | | |
| --- | --- | --- | --- |
| **Score** | | **Category** | **Description** |
| 1 | Death | | Patient did not survive· |
| 2 | Persistent vegetative state | | No obvious cortical function; patient is unresponsive and unable to interact with the environment· |
| 3 | Severe disability | | Patient is conscious but dependent on others for daily support due to physical or mental disability· |
| 4 | Moderate disability | | Patient is independent but has residual deficits such as neurological or cognitive impairment· |
| 5 | Good recovery | | Patient has resumed normal activities, although minor neurological or psychological deficits may persist· |

| **Supplementary table 2** Factors associated with unfavourable outcome in patients with pneumococcal meningitis | | | | | |
| --- | --- | --- | --- | --- | --- |
|  | **Favourable outcome**  N = 1,184 | **Unfavourable outcome**  N = 845 | **Univariable odds ratio for unfavourable outcome** | **Multivariable odds ratio for unfavourable outcome** | **p value of multivariable analysis** |
| Age (years) | 60 (49-67) | 65 (56-74) |  |  | **<0·001**^†^ |
| 16-39 | 156/1,184 (13%) | 52/845 (6%) | *Reference* | *Reference* |  |
| 40-70 | 852/1,184 (72%) | 499/845 (59%) | 1·76 (1·27-2·47) | 1·32 (0·89–1·94) | 0·165 |
| **>70** | 176/1,184 (15%) | 294/845 (35%) | 5·01 (3·50-7·27) | 3·42 (2·21–5·28) | **<0·001** |
| **Symptoms <24h** | 610/1,154 (53%) | 343/787 (44%) | 0·69 (0·57-0·83) | 0·76 (0·61–0·95) | **0·016** |
| **Otitis or sinusitis** | 612/1,163 (53%) | 284/799 (36%) | 0·50 (0·41-0·60) | 0·77 (0·61–0·96) | **0·023** |
| **Pneumonia** | 93/1,156 (8%) | 144/794 (18%) | 2·53 (1·92-3·35) | 1·55 (1·11–2·18) | **0·011** |
| Immunosuppressive drug use | 84/1,178 (7%) | 65/835 (8%) | 1·10 (0·78-1·54) | 0·88 (0·57–1·35) | 0·550 |
| Splenectomy | 26/1,180 (2%) | 25/844 (3%) | 1·35 (0·77-2·37) | 1·28 (0·66–2·46) | 0·464 |
| Active cancer | 140/1,182 (12%) | 131/844 (16%) | 2·01 (1·38-2·96) | 1·23 (0·76–1·99) | 0·403 |
| **Diabetes Mellitus** | 143/1,178 (12%) | 148/837 (18%) | 1·55 (1·21-2·00) | 1·51 (1·10–2·08) | **0·010** |
| **Alcoholism** | 51/1,178 (4%) | 90/839 (11%) | 2·66 (1·87-3·81) | 1·98 (1·29–3·05) | **0·002** |
| Known HIV | 6/1,181 (1%) | 6/842 (1%) | 1·41 (0·44-4·51) | 1·57 (0·38–6·47) | 0·536 |
| Antibiotics before admission | 133/1,157 (11%) | 63/826 (8%) | 0·64 (0·46-0·87) | 0·75 (0·51–1·10) | 0·145 |
| Headache | 889/1,070 (83%) | 446/615 (73%) | 0·54 (0·42-0·68) | 0·90 (0·67–1·20) | 0·468 |
| Nausea | 584/1,003 (58%) | 315/614 (51%) | 0·76 (0·62-0·92) | 0·96 (0·75–1·23) | 0·765 |
| Neck stiffness | 827/1,085 (76%) | 524/751 (70%) | 0·72 (0·58-0·89) | 0·82 (0·59–1·14) | 0·229 |
| Rash | 34/1,038 (3%) | 28/723 (4%) | 1·19 (0·71-1·98) | 1·28 (0·71–2·32) | 0·412 |
| **Heart rate (b/min)** | 99 (84-111) | 104 (89-120) | 1·01 (1·01-1·02) | 1·01 (1·00–1·01) | **<0·001** |
| Diastolic BP (mmHg) | 80 (70-90) | 80 (70-94) |  |  | 0·415^†^ |
| <60 | 93/1,145 (8%) | 84/805 (10%) | 0·72 (0·52-0·98) | 1·29 (0·89–1·87) | 0·186 |
| 60-100 | 938/1,145 (82%) | 606/805 (75%) | Reference | Reference |  |
| >100 | 114/1,145 (10%) | 115/805 (14%) | 1·12 (0·75-1·66) | 1·02 (0·73–1·42) | 0·922 |
| **Temperature (°C)** | 39·00 (38·20-39·70) | 38·60 (37·50-39·50) | 0·78 (0·73-0·83) | 0·86 (0·78–0·96) | **0·006** |
| **GCS score** | 11 (9-13) | 10 (8-12) | 0·85 (0·83-0·88) | 0·88 (0·84–0·92) | **<0·001** |
| Triad^*^ | 531/1,108 (48%) | 321/777 (41%) | 0·76 (0·64-0·92) | 0·85 (0·60–1·20) | 0·350 |
| **Cranial nerve palsy** | 60/997 (6%) | 88/669 (13%) | 2·37 (1·68-3·35) | 2·03 (1·34–3·07) | **<0·001** |
| **Seizures** | 84/1,154 (7%) | 118/785 (15%) | 2·25 (1·68-3·04) | 1·73 (1·20–2·50) | **0·003** |
| Focal neurological deficits | 245/1,090 (22%) | 214/692 (31%) | 1·54 (1·25-1·91) | 1·26 (0·98–1·62) | 0·071 |
| **CRP – blood (mg/L)** ^§^ | 161 (76-260) | 261 (141-370) | 1·00 (1·00-1·01) | 1·03 (1·02–1·04) | **<0·001** |
| Thrombocyte count | 212 (162-267) | 187 (138-251) |  |  | 0·071^†^ |
| <150 | 218/1,133 (19%) | 250/795 (31%) | 0·52 (0·42-0·64) | 1·25 (0·96–1·62) | 0·092 |
| 150-450 | 882/1,133 (78%) | 526/795 (66%) | *Reference* | *Reference* |  |
| >450 | 33/1,133 (3%) | 19/795 (2%) | 0·50 (0·27-0·90) | 0·60 (0·29–1·23) | 0·165 |
| CSF WBC count (cells/mm3) | 3,017 (971-7,344) | 1,367 (179-5,494) |  |  |  |
| **< 100** | 73/1,163 (6%) | 157/813 (19%) | 0·53 (0·42-0·66) | 2·20 (1·50–3·24) | **<0·001** |
| **100-999** | 225/1,163 (19%) | 215/813 (26%) | 2·25 (1·62-3·16) | 1·48 (1·12–1·94) | **0·006** |
| 1000-9999 | 668/1,163 (57%) | 337/813 (41%) | *Reference* | *Reference* |  |
| >10 000 | 197/1,163 (17%) | 104/813 (13%) | 0·55 (0·41-0·75) | 0·91 (0·66–1·25) | 0·542 |
| **CSF:blood glucose ratio** | 0·05 (0·01-0·27) | 0·02 (0·01-0·09) | 0·09 (0·04-0·17) | 0·31 (0·14–0·66) | **0·003** |
| **CSF protein (g/L)** | 3·7 (2·3-5·9) | 4·9 (3·2-7·3) | 1·12 (1·09-1·16) | 1·08 (1·04–1·12) | **<0·001** |
| Positive blood culture | 865/1,044 (83%) | 605/727 (83%) | 1·03 (0·80-1·32) | 0·97 (0·71–1·31) | 0·829 |
| ^*^ Triad of fever, neck stiffness and altered mental status ^§^ Odds ratios for CRP are expressed per 10mg/L increase  ^†^ Overall p-values for categorical variables with more than two categories were calculated using the Likelihood Ratio Test (LRT) from the multivariable logistic regression model.  Data are shown as median [IQR] or n/N (%). Odds ratios are provided with 95% CI· The multivariable analysis used an imputed dataset with 50 imputation sets with 10 iterations per set. All variables in the table were entered in the multivariable regression model simultaneously. Bold font indicates statistically significant results in the multivariable regression. Abbreviations: HIV = human immunodeficiency virus, GCS=Glasgow Coma Scale, CSF=cerebrospinal fluid, WBC = white blood cell count, CRP = C-reactive protein. | | | | | |
